# The Influence of Welding Process Parameters on Pore Formation in Pulsed Laser-Welded Vacuum Plate Glazing

**DOI:** 10.3390/ma12111790

**Published:** 2019-06-02

**Authors:** Shanwen Zhang, Chong Li, Hong Miao, Qiang He

**Affiliations:** College of Mechanical Engineering, Yangzhou University, Yangzhou 225127, China; Licyzu0905@163.com (C.L.); jixieheqiang@163.com (Q.H.)

**Keywords:** pore formation, laser-welded, vacuum plate glazing, porosity, theoretical basis

## Abstract

To explore the mechanism of the pore formation in the laser-welded vacuum plate glazing sealing layer, a vacuum plate glazing laser side sealing test was carried out. The influence of the pulse current, pulse duration time, pulse frequency and welding speed on the pores of the sealing layer was studied by means of scanning electron microscopy (SEM) and metallographic microscopy, and the cause of the pores is analyzed. The results show that the porosity decreases with the increase of the pulse current. When the pulse current is 160 A, the pores are the fewest and smallest, and the porosity is only 0.1%. The porosity of the sealing layer decreases first and then increases with the increase of the pulse duration time. The porosity decreases first and then increases with the increase of the pulse frequency. When the pulse frequency is 18 Hz, the porosity is the least, at only 0.08%. The porosity of the sealing layer increases with the increase of the welding speed. When the welding speed is 80, 90, 100 and 110 mm/min, the porosity is 0.02, 0.08, 0.63 and 0.89%, respectively. These studies can provide a theoretical basis for laser sealing manufacturing of vacuum plate glazing.

## 1. Introduction

Vacuum glazing has the characteristics of low heat transfer coefficient, a high dew resistance factor level, high sound insulation performance, long life and light structure. It has become an important energy-saving thermal insulation material, which has been widely used in construction, agriculture and other fields such as solar water heater, thermal insulation cabinet and so on [1]. The reliable edge sealing of vacuum glazing is the first step and a necessary prerequisite for its successful application [2,3]. The traditional method of vacuum glazing, heating furnace edge-sealing is time-consuming, energy-consuming, has poor adaptability to glazing size, and cannot meet the dependence of vacuum glazing on tempering, which limits the development of vacuum glazing industrialization. The search for a "no-heating furnace" edge-sealing technology has become a research hotspot in this field.

In 2014, Kim et al. [4] studied the effect of process parameters on the bending strength and shape of glazing by using the gas torch for vacuum glazing edge sealing experiments, and measured the sealing strength of the edge of vacuum glazing using the Taguchi test method, and determined the error rate of the two by comparing the experimental value with the predicted value. In 2015, Menon, et al. [5] developed a low temperature (below 200 °C) composite Cerasolzer CS186 alloy (Sn (56%), Pb (39%), Zn (3%), Sb (1%), A1-Ti-Si-Cu (1%)) as the surrounding encapsulation material for vacuum glazing (lead-tin alloy edge-sealing), and three-layer vacuum glazing was sealed by ultrasonic welding technology. SEM and X-ray diffraction were used to study the properties of Cerasolzer micro-structural glazing and Cerasolzer interface joint, and the mechanical bonding properties of the edge sealing were analyzed. A finite element model of 300 mm × 300 mm three-layer vacuum glazing was established to study the effect of the edge seal width on the thermal performance during heat transfer. The results showed that the central plate and the total thermal transmittance value are 0.33 and 1.05 W/(m^2^·K), respectively. Appropriately reducing the edge sealing width is beneficial to improving the heat transfer performance of vacuum glazing. However, due to the high price of lead-tin alloy and the high cost of sealing material, this technology has not yet entered the industrialization stage. In 2018, Young et al. [6] studied the effect of edge seal shape on the characteristic strength of vacuum glazing. According to the shape and size parameters of a Weibull distribution, the characteristic strength and failure distribution were analyzed. The failure probability and the acceptable seal shape were determined according to relevant criteria. In 2018, Zhang et al. [7] used scanning electron microscopy (SEM) and energy spectrum analysis (EDS) to analyze the micro-structure of the sealing layer and studied the mechanism of formation of the sealing hole. The results showed that the sealing layer has a uniform distribution in the two-layer structure of the wetting layer, and there are a large number of isolated pores which are not connected to each other. With the increase of laser power, the interface layer becomes compact and smooth, and the number of pores is reduced. When the power exceeds a certain value, the interface becomes rough with the increase of the number of pores. The faster the welding speed is, the smaller the energy output is, forming a block structure, with unmelted solder, pores and cracks.

Laser welding has the advantages of good connection quality, high degree of automation, environmental protection and cleanliness, and high economic benefits, etc., which can effectively compensate for the shortcomings of heating furnace edge sealing technology. Therefore, the laser sealing vacuum flat glazing has been systematically studied by means of experiments [8,9,10]. This study analyzes the pore distribution and the porosity variation trends of the sealing layer under different welding parameters through single factor control variable method. A set of ideal experimental parameters for laser sealing are thus obtained.

## 2. Materials and Methods

### 2.1. Experimental Preparation

**Figure 1:**
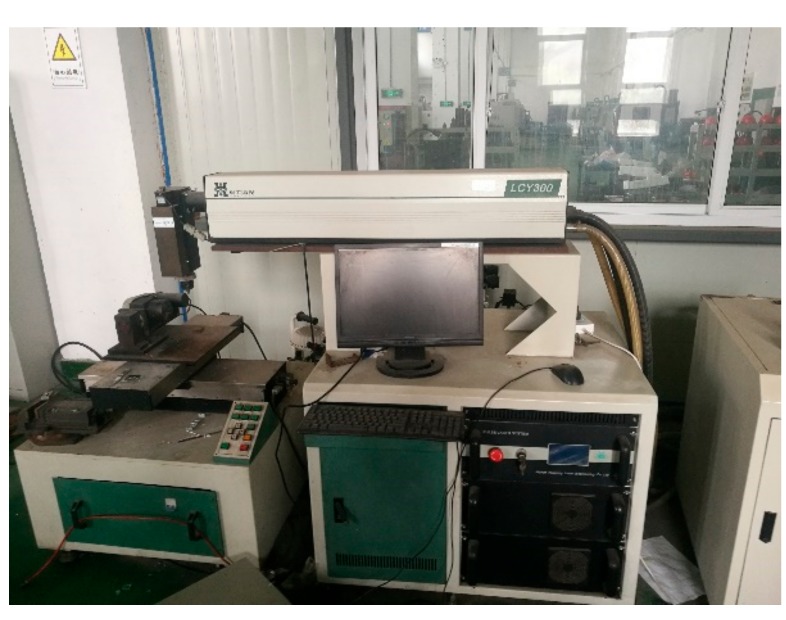
HGL-LCY300 type Nd:YAG laser welding system.

The size of the soda-lime glazing used in our welding experiments is 50 mm × 50 mm × 4 mm, and its chemical composition is shown in Table 1. Before welding, the surface of the glazing is smooth, and the roughness is too small to meld with solder, so the glazing surface is polished by a BD46N abrasive belt grinder (Dongguan Shijie Hongsheng Hardware Store, Dongguan, China), which imparts a makes certain roughness to the glazing. Because of dirt such as dust and oil stains on the glazing surface, it needs to be cleaned by ultrasound in absolute ethanol for 10 min, then dried in a DHG-9053A type electrothermal constant temperature blast dryer (Shanghai Shenxian Heat Constant Equipment Factory, Shanghai, China), and the prepared solder is coated around the flat glazing. Heating is carried out on the D20 digital display heating board at 150 °C for 30 min to eliminate the adhesive and avoid the defects of air holes and bubbles in the sealing part. Then it is combined with another piece of glazing and preheated at 250 °C for 10 min. Finally, the experimental samples were placed on the HGL-LCY300 Nd: YAG laser platform (Wuhan Huagong Laser Engineering Company, Wuhan, China), and obtain vacuum plate glazing samples under various process parameters [11]. The HGL-LCY300 type Nd: YAG laser welding system is shown in Figure 1, and the laser process parameters used in the test are shown in Table 2.

### 2.2. Experimental Materials

The solder used in the experiment is PbO-TiO_2_-SiO_2_-R*_x_*O*_y_* and its main components are listed in Table 3.

Since the solder is a powder it is difficult to coat on the glazing surface, so solders need to be converted into a liquid slurry before use. Here, we use a mixed solution of ethyl cellulose and terpineol as blending agent. The preparation process is as follows: ethyl cellulose and terpineol with a mass ratio of 1:9 are put into a beaker, mixed and stirred until the ethyl cellulose is completely dissolved under water bath heating at 80 °C. Then the solder and solvent with a mass ratio of 10:1 are mixed, stirred for 30 min and then defoaming is performed for 30 min to complete the preparation of the coated solder [12].

### 2.3. Experimental Testing Instrument

#### 2.3.1. Metallographic Analysis

**Figure 2:**
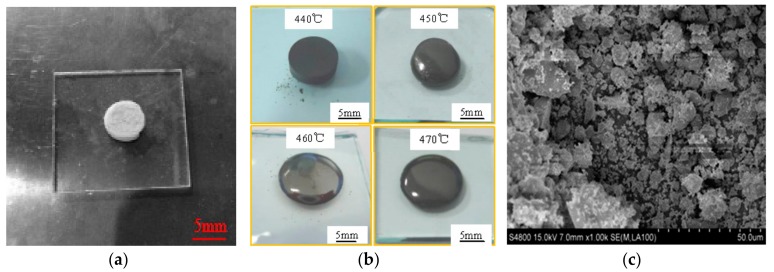
Experimental materials: (**a**) the glazing; (**b**) the solder shape at different temperatures; (**c**) microstructure of the sealing solder under scanning electron microscope (SEM).

**Figure 3:**
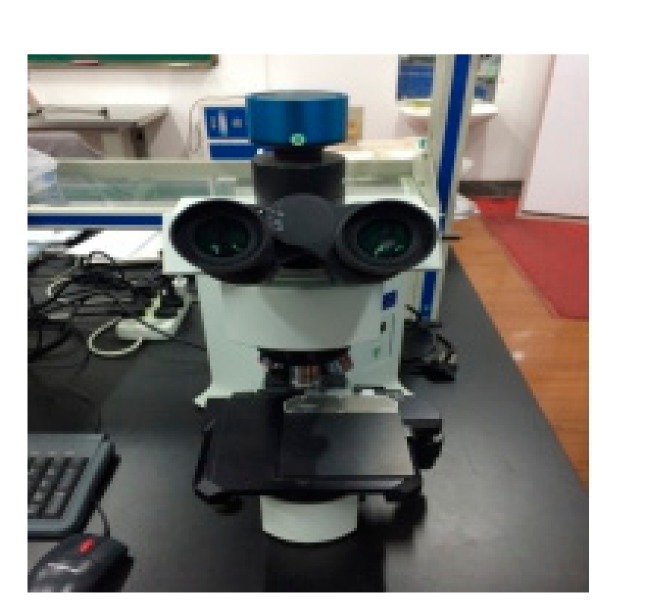
BX41M-LED metallographic microscope.

We use a BX41M-LED metallographic microscope (Shanghai Wickham Photoelectric Technology Company, Shanghai, China) to observe the bonding between solder and glazing at the joint interface under different process parameters, and analyse the relationship between the interface reaction wetting layer and the process parameters. Then we observe the pores and distribution characteristics by the metallographic microscope, and use the Image-J software (Image-J 1.48, National Institutes of Health, New York, NY, USA) to calculate the porosity of the sealing layer after metallographic photograph processing. The porosity is the percentage of the pore area to the surface area of the weld. The porosity *p_r_* is a percentage of the pore area *A_p_* and the area of the upper surface of the weld *A_w_*, i.e., *p_r_* = (*A_p_*/*A_w_*) × 100%. The particle size of the sealing solder used in the experiment ranges from 1 to 25 μm with an average particle size of 12.15 μm. Figure 2a shows the glazing and solder used in the experiment, Figure 2b shows the solder shape at different temperatures, and Figure 2c shows the microstructure of sealing solder under scanning electron microscope (SEM). Figure 3 is the BX41M-LED metallographic microscope.

#### 2.3.2. Micromorphology and Phase Analysis

**Figure 4:**
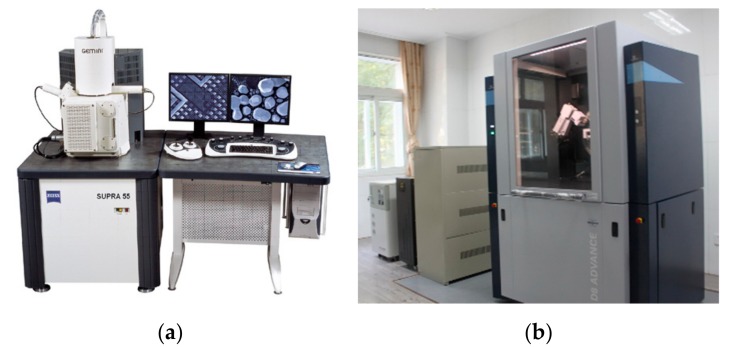
The test instruments: (**a**) the Zeiss Supra 55 field emission scanning electron microscope; (**b**) the D8-Advance type crystal X-ray diffractometer.

We used a Zeiss Supra 55 field emission scanning electron microscope (Hitachi High-Technologies Corporation, Tokyo, Japan) to observe the microstructure of the molten layer under different process parameters, and then used the self-contained energy spectrometer to perform line scan analysis on the reactive wetting layer. Finally, we carried out sample analyses with the D8-Advance type crystal X-ray diffractometer (Germany Bruker-AXS, Heidelberg, Germany). The test instrument is shown in Figure 4.

## 3. Results and Discussion

### 3.1. Pore Source and Formation Reasons

**Figure 5:**
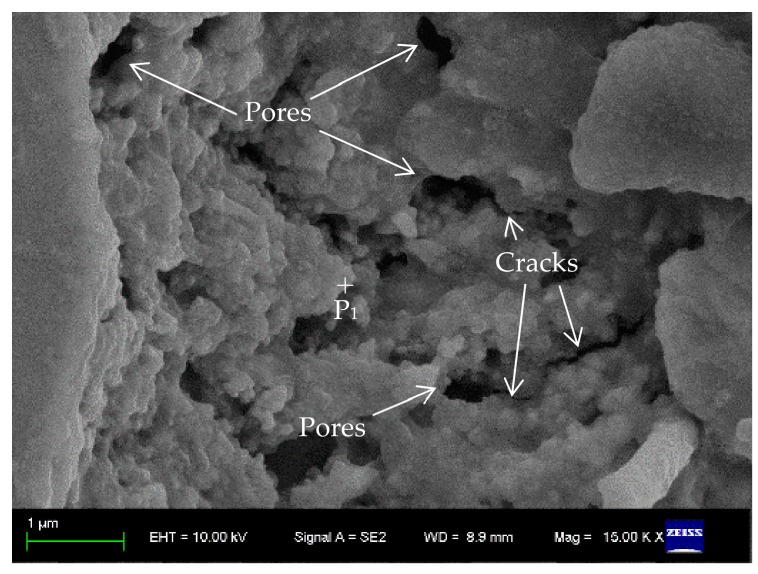
Microstructure of sealing layer under scanning electron microscope (SEM) (*P* = 80 W, *v* = 2 mm/s).

The presence of gas is a prerequisite for pore generation. There are four main sources of gas in the welding process: residual air between powder particle gaps or on the substrate surface, powder or matrix containing a certain amount of organic matter, powder or volatilization of low melt metal compounds in the matrix and chemical reactions of the powder during the melting process that produce the new gas. To reveal the reason for the pore formation in the sealing layer during the laser sealing process, this study grouped the samples and welded them with different process parameters, and took out the sample, which was obtained by *P* = 80 W and *v* = 2 mm/s, to be subjected to SEM analysis, as shown in Figure 5.

A large number of isolated pores with different sizes and disconnections occur in the sealing layer, mainly located at the particle junctions. Micro-cracks appear at the edges of some pores, and the cracks extend from the pores to the molten mass. In the vacuum plate glazing sealing process, due to the differences in thermal expansion coefficients of the solder and the glazing substrate, which generates a certain thermal stress in the sealing layer. Moreover, the pores will also generate a certain pressure increase as the heat rises during the formation process. Under the joint action of the two phenomena, the cracks are preferentially generated at the pores, and the crack generation may easily connect some isolated pores, which affects the sealing quality of the vacuum plate glazing.

**Figure 6:**
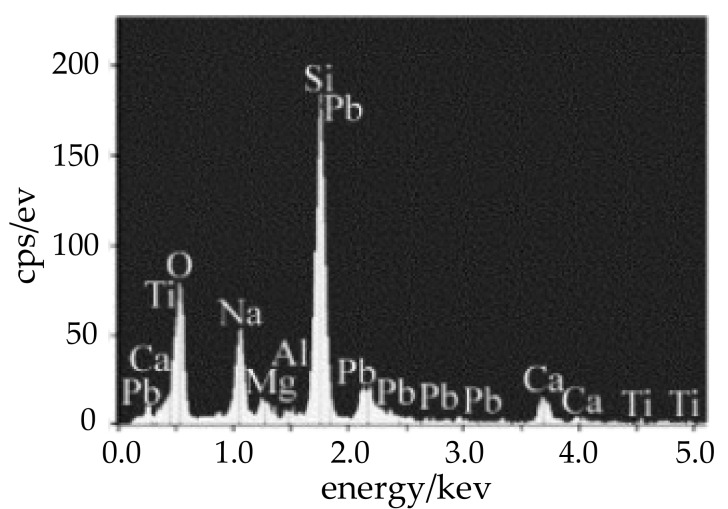
Energy spectrum diagram of *P*_1_ in Figure 2.

Figure 6 shows the energy spectrum diagram of *P*_1_ in Figure 5, and it shows that the main elements of the sealing layer are Si, Pb, Ti, Na, O, etc. It does not present unique organic matter elements such as C, S in the sealing layer, so we can discount organic matter as the cause of the pores. The main solder components are PbO, TiO_2_, SiO_2_, Cuo and Fe_2_O_3_, which are all hardly volatile materials, so pores formation caused by the volatilization of the solder and its additives can be eliminated.

Table 4 shows EDS analysis results for each region. The main components of the sealing layer are Pb, Ti, O, Si, O. The main components of glazing include Si, O, and some additive elements like Ca, Na and Mg, etc.

**Figure 7:**
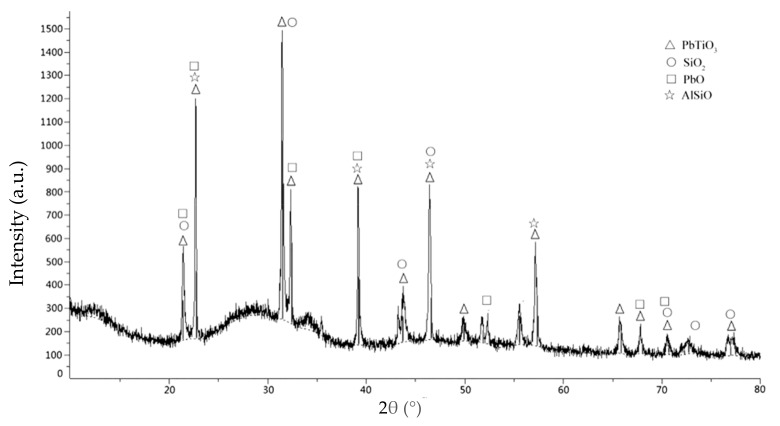
The XRD analysis results of the sealing layer.

In the reaction wetting layer, the elements such as Pb, Ti, O, Si, Ca, Na and Al migrate to form a component transition zone. Figure 7 shows the XRD analysis results of the sealing layer. Results show that the crystal phase formed during laser welding is mainly PbTiO_3_, which also contains some PbO and SiO_2_ phases. PbO and TiO_2_ react chemically to form PbTiO_3_, but no gas is formed during this process, so this can be eliminated as the cause of the pores caused by the powder reaction.

In addition, since the solder used in the experiment is the PbO-TiO_2_-SiO_2_-R*_x_*O*_y_* system sealing solder, the solder is a low melting point solder. During the soldering process, the soldering temperature is generally 25 to 60 °C higher than the melting temperature of the solder, and the excess temperature causes a small amount of evaporation of the solder. In summary, the cause of the pores in laser welded vacuum plate glazing is probably residual air and steam between the solder powders.

### 3.2. Effect of Pulse Current on Sealing Layer Pores

When the pulse duration time τ is 2 ms, the pulse frequency *f* is 15 Hz, the welding speed *v* is 90 mm/min, and the pulse current is changed, Table 2 shows the welding process parameters when the pulse current is changed.

**Figure 8:**
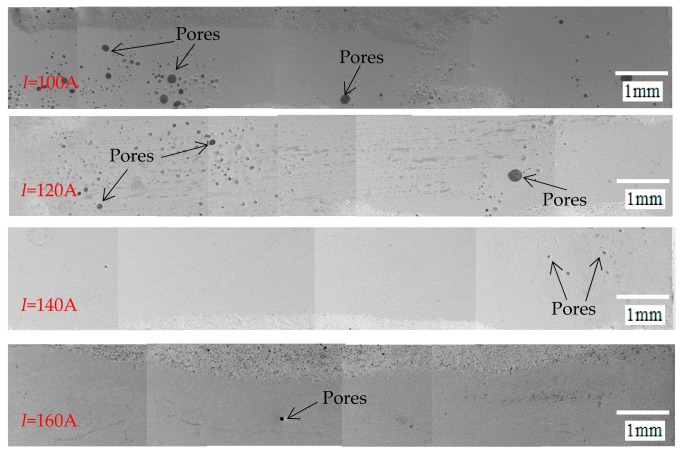
The pore distribution under different pulse currents.

**Figure 9:**
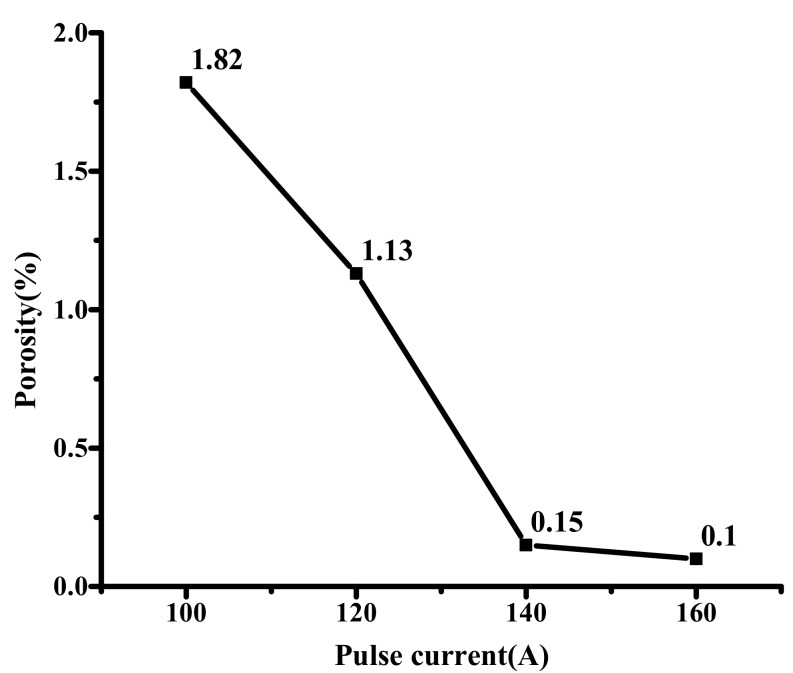
The porosity variation curve of the sealing layer under different pulse currents.

Figure 8 shows the pore distribution under different pulse currents. The results show that the porosity decreases with the increase of the pulse current. When the pulse current is 100 and 120 A, the number of pores in the sealing layer is large and widely distributed, and the porosity is 1.82% and 1.13%, respectively. When the pulse current is 140 and 160 A, the pores are less and small, and the porosity is only 0.15% and 0.1%, respectively. In combination with Figure 9, this is because when the pulse current is low, the laser input energy is small, so the solder is not completely melted, and the amount of liquid phase is small. During the cooling process, the joint cools rapidly, and the melt viscosity increases rapidly. As a result, the bubble volume decreases, and density increases, which causezs the bubbles to float slower, so many bubbles do not escape in time and remain inside to form pores. When the pulse current increases, the amount of liquid phase increases, the viscosity of the melt becomes lower, and the convection of the solder is enhanced, which helps the residual gas between the solder particles to condense into a large bubble at a high temperature and then discharge. Therefore, larger current can obtain the sealing layer with lower porosity.

### 3.3. Effect of Pulse Duration Time on Sealing Layer Pores

When the pulse current *I* is 160 A, the pulse frequency *f* is 15 Hz, the welding speed *v* is 90 mm/min, and the pulse duration time is changed, Table 2 shows the welding process parameters when pulse duration time is changed.

**Figure 10:**
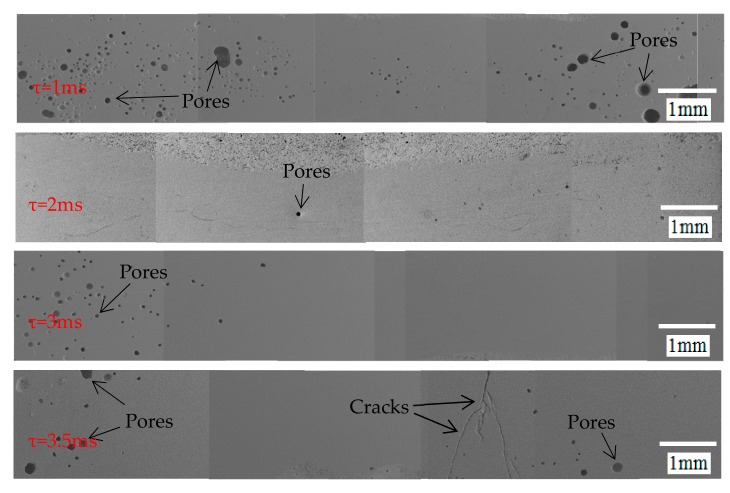
The pores distribution under different pulse duration times.

**Figure 11:**
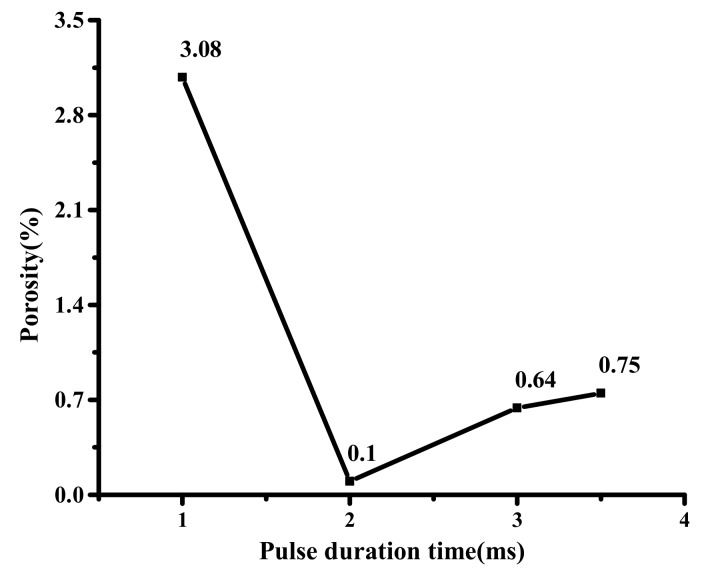
The porosity variation curve of the sealing layer under different pulse duration times.

Figure 10 shows the pores distribution under different pulse duration times. The results show that the number of the pores decreases first and then increases. This is because when the pulse duration time is 1 and 2 ms, as the pulse duration time increases, the heat source acts longer, prolonging the relative thermal action time of the liquid solder convection, so the bubbles have enough time to escape from the sealing layer. However, the excessively large pulse duration time will cause the solder to have a higher temperature than other conditions, resulting in a small amount of solder vapor inside the sealing layer, which may be a cause of the porosity increase of the pulse duration time at 3 and 3.5 ms. At the same time, when the pulse duration time is 3 and 3.5 ms, due to the occurrence of “spheroidization” effect [13], grain coarsening during the cooling process leads to an increase in melt viscosity and is not conducive to gas evolution. When the pulse duration time is 1 ms, the number of pores in the sealing layer is large and widely distributed, and the porosity is 3.08%. When the pulse duration time is 2 ms, the pores are the least, and the porosity is only 0.1%. When the pulse duration time is 3 and 3.5 ms, the pores become less and small, and the porosity is only 0.64% and 0.75% respectively. Figure 11 indicates the porosity variation curve of the sealing layer under different pulse duration times.

### 3.4. Effect of Pulse Frequency on Sealing Layer Pores

When the pulse current *I* is 160 A, the pulse duration time τ is 2 ms, the welding speed *v* is 90 mm/min, and the pulse frequency is changed, Table 2 includes the welding process parameters when the pulse frequency is changed.

**Figure 12:**
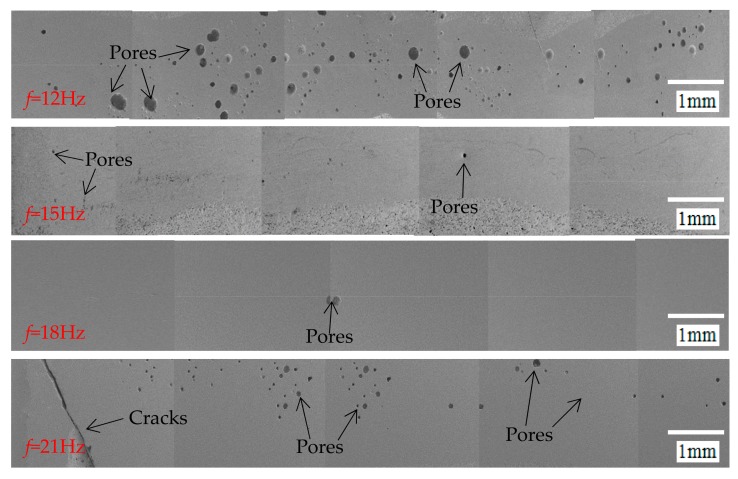
The pores distribution under different pulse frequencies.

**Figure 13:**
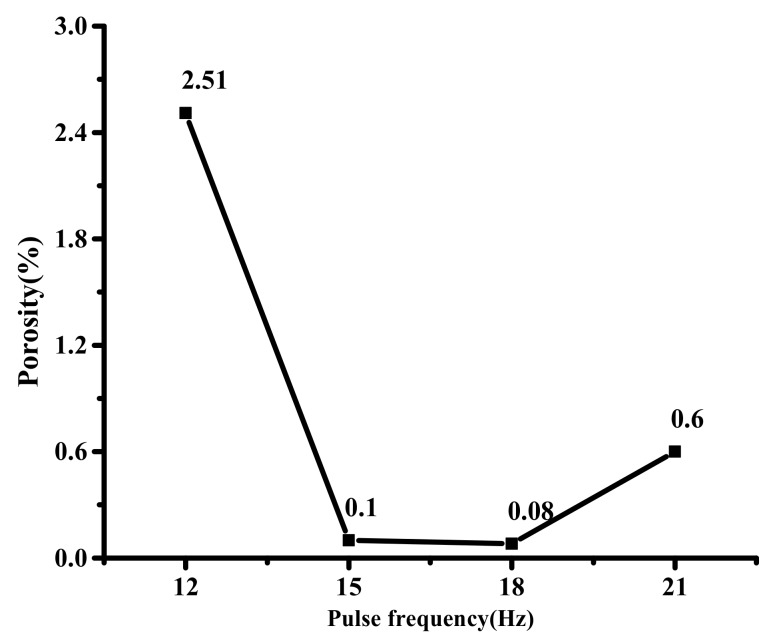
The porosity variation curve of the sealing layer under different pulse frequencies.

The pore distribution under different pulse frequencies is shown in Figure 12. When the pulse frequency is 12 Hz, the number of pores in the sealing layer is large and widely distributed, and the porosity is 2.51%. When the pulse frequency is 15 and 18 Hz, respectively, the pores are fewer and small. When the pulse frequency is 18 Hz, the porosity is the least, only 0.08%. When the pulse frequency is 21 Hz, the number of pores is increasing, and the porosity is 0.6%. Figure 13 shows the porosity variation curve of the sealing layer under different pulse frequencies. Matsunawa [14,15] believed that during the pulse laser welding process, the small pores are strongly evaporated locally, and the dynamic pressure of the steam is equivalent to a stirring force to the molten pool, promoting liquid solder flow. The larger the pulse frequency, the more obvious the stirring effect on the sealing layer. When the pulse frequency is small, the dynamic pressure of the steam is insufficient to allow the liquid solder to flow rapidly, generating in the sealing layer. The pulse frequency will make the steam dynamic pressure large and the steam more, which is easy to form pores.

When the pulse frequency is 21 Hz, there are many micro-cracks on the molten layer surface. The cause of the cracks may be that the laser inputs excessive energy, which melts the solder completely, so the sealing layer is flat and dense after solidification. However, the liquid solder shrinks during the cooling process and generates the tensile stress in the sealing layer. The tensile stress pulls the solidified weld and forms a crack when there is not enough liquid solder to replenish. So when the pulse frequency is 18 Hz, the frequency at this time is the most ideal value in the test. When the following test involves frequency, the frequency value is taken as 18 Hz.

### 3.5. Effect of Welding Speed on Sealing Layer Pores

When the pulse current *I* is 160 A, the pulse duration time τ is 2 ms, the pulse frequency *f* is 18 Hz, and the welding speed is changed, Table 2 indicates the welding process parameters when the welding speed is changed.

**Figure 14:**
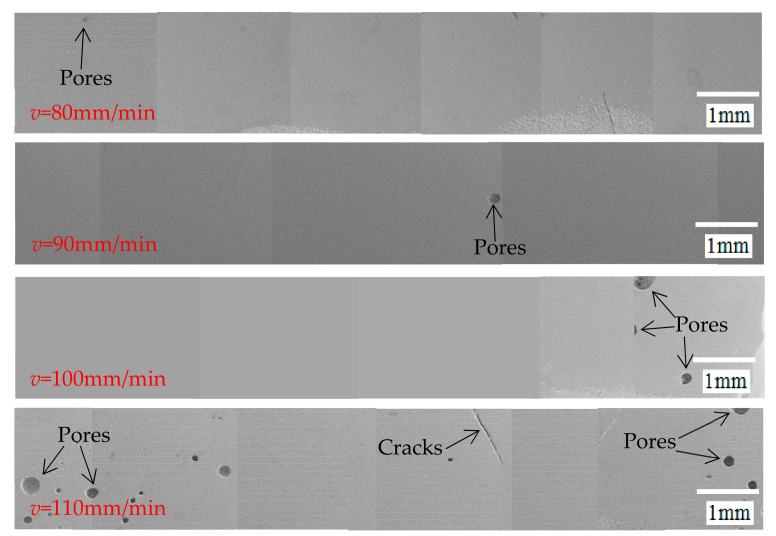
The distribution of pores at different welding speeds.

**Figure 15:**
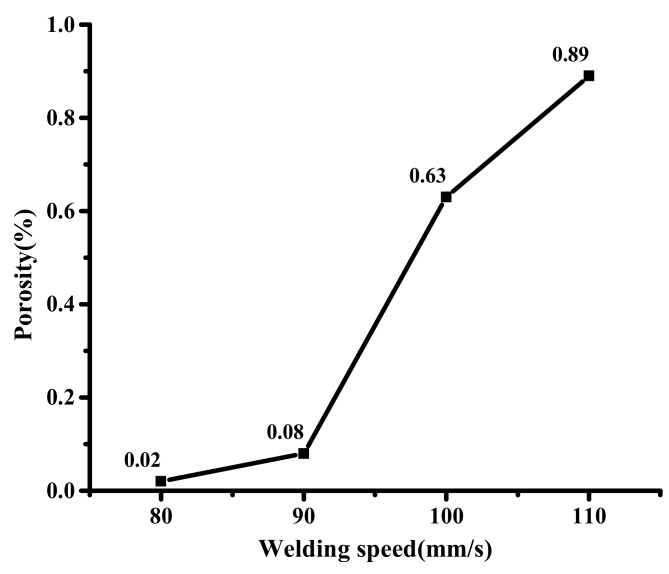
The porosity variation curve of the sealing layer at different welding speeds.

Figure 14 is a graph showing the pore distribution at different welding speeds. Results show that the number of pores increases with the increase of the welding speed. On the one hand, the temperature gradient of the sealing layer increases with the increase of the welding speed, which accelerates the cooling rate of the molten solder, and makes the bubbles solidify quickly. On the other hand, as the welding speed increases and the melt viscosity increases, it will hinder the escape of the bubbles thus forming pores. The porosity variation curve of the sealing layer at different welding speeds is shown in Figure 15. The number of pores in the sealing layer is larger and larger and widely distributed with the increase of welding speed. When the welding speed is 80, 90, 100 and 110 mm/min, the porosity is 0.02, 0.08, 0.63 and 0.89%, respectively.

## 4. Conclusions

This study first introduces the HGL-LCY300 type Nd:YAG laser welding system as the experimental platform, the experimental materials and method of laser sealing vacuum plate glazing. Then we use a single factor control experiment to study of the influence of pulse current, pulse duration time, pulse frequency and welding speed on the pores distribution and porosity variation of sealing layer. The experimental results can be summarized as follows:

(1) The pores in the laser welded vacuum plate glazing are probably caused by the residual air and steam between the solder powders.
(2) The porosity decreases with the increase of the pulse current. When the pulse current is 160 A, the porosity is at least, at 0.1%. The porosity of the sealing layer decreases first and then increases. When the pulse duration time is 2 ms, the porosity is the lowest. The porosity decreases first and then increases. When the pulse frequency is 18 Hz, the lowest porosity is 0.08%. When the welding speed is increased, the joint cooling rate is higher than the air hole escape speed, resulting in the number of the sealing layer pores increasing.
(3) According to the porosity variation curve of the sealing layer under different welding parameters, the slope formula is used to calculate the influence level on porosity under different welding parameters. Results show that the variation of pulse duration times has the greatest influence on the porosity, the influence of pulse frequency on porosity is the second, and the variations of pulse current and welding speed have the slight influence on the porosity. Therefore, the influence level on the porosity under different welding parameters is as follows: pulse duration times > pulse frequency > pulse current ≈ welding speed.
(4) When the pulse current *I* is 160 A, pulse duration time τ is 2 ms, pulse frequency *f* is 18 Hz and welding speed *v* is 90 mm/min, the sealing layer has low porosity and good welding quality, which represents an optimal set of process parameters.

## Figures and Tables

**Table 1:** Chemical composition of the common soda-lime glazing (mass fraction (%)).

Chemical Composition	Mass Fraction (%)
SiO_2_	0.67–0.74
CaO	0.05–0.11
MgO	0–0.04
Na_2_O	0.10–0.17
Al_2_O_3_	0–0.03

**Table 2:** The laser process parameters used in the tests.

Pulse Current (A)	Pulse Duration Time (ms)	Pulse Frequency (Hz)	Welding Speed (mm/min)	Single Pulse Energy (J)	Average Power (W)
100	2	15	90	2.28	34.2
120	2	15	90	2.74	41.1
140	2	15	90	3.19	47.9
160	2	15	90	3.65	54.8
160	1	15	90	1.82	27.4
160	2	15	90	3.65	54.8
160	3	15	90	5.47	82.1
160	3.5	15	90	6.38	95.8
160	2	12	90	3.65	43.8
160	2	15	90	3.65	54.8
160	2	18	90	3.65	65.7
160	2	21	90	3.65	76.6
160	2	18	80	3.65	65.7
160	2	18	90	3.65	65.7
160	2	18	100	3.65	65.7
160	2	18	110	3.65	65.7

**Table 3:** Chemical composition of the sealing solder (mass fraction (%)).

Chemical Composition	Mass Fraction (%)
PbO	0.75
TiO_2_	0.15
SiO_2_	0.10
CuO	<0.025
Fe_2_O_3_	<0.025

**Table 4:** EDS analysis results for each region (at.%).

Area	Pb	Ti	O	Si	Na	Ca	Al	Mg	Fe	K	Cu
Sealing layer	21.02	16.56	60.55	0.72	0.17	0.13	0.04	-	0.41	-	0.40
Glazing	15.68	4.25	69.11	7.88	0.30	0.21	0.28	-	1.38	0.12	0.61
Reaction wetting layer	0.08	0.03	59.23	24.77	9.22	3.95	0.34	1.97	0.07	0.33	-

## References

[1] Kim J.K., Kim Y.S., Jeon E.S. (2019). Optimization of glazing edge sealing process using microwaves for fabrication of vacuum glazing. Appl. Sci..

[2] Lee W., Kang J., Cho S.W. (2018). A new structure of vacuum insulation glazing for edge effect reduction: A parametric study. Int. J. Precis. Eng. Manuf..

[3] Memon S., Farukh F., Kandan K. (2018). Effect of cavity vacuum pressure diminution on thermal performance of triple vacuum glazing. Appl. Sci..

[4] Kim S.J., Jeon E.S. (2014). Analysis of the edge sealing strength for vacuum glazing panel using design of experiment. J. Korea Acad.-Ind. Coop. Soc..

[5] Memon S., Farukh F., Eames P.C., Silberschmidt V.V. (2015). A new low-temperature hermetic composite edge seal for the fabrication of triple vacuum glazing. Vacuum.

[6] Kim Y.S., Jeon E.S. (2018). Using a hydrogen gas torch to seal edges of vacuum glazing panels and analysis of the related characteristic strength according to the sealed edge shapes. Vacuum.

[7] Zhang J., Liu S., Zhang Y., Miao H., Zhang S., Zhang Q. (2018). Formation mechanism of sealing edge pores for vacuum glazing using laser brazing technique. Vacuum.

[8] Memon S., Fang Y., Eames P.C. (2019). The influence of low-temperature surface induction on evacuation, pump-out hole sealing and thermal performance of composite edge-sealed vacuum insulated glazing. Renew. Energy.

[9] Miao H., Shan X., Zhang J., Sun J., Wang H. (2015). Effect of sealing temperature on the sealing edge performance of vacuum glazing. Vacuum.

[10] Zhao J.F., Eames P.C., Hyde T.J., Fang Y., Wang J. (2007). A modified pump-out technique used for fabrication of low temperature metal sealed vacuum glazing. Sol. Energy.

[11] De Pablos-Martín A., Höche T. (2017). Laser welding of glasses using a nanosecond pulsed Nd:YAG laser. Opt. Lasers Eng..

[12] Li Y., Tian R., Xiao Y., Wang W., Ding X., Yin L., Zhang J. (2017). Improved the quality of the glass/glass laser bonding through the optimization of glass powder size in planetary ball mill. Mol. Cryst. Liq. Cryst..

[13] Gu D.D., Shen Y.F., Pan Y.F., Xu C.T. (2004). Research on mechanism of direct metal laser sintering. J. Mater. Eng..

[14] Matsunawa A., Semak V. (1997). The simulation of front keyhole wall dynamics during laser welding. J. Phys. D Appl. Phys..

[15] Matsunawa A., Kim J.-D., Seto N., Mizutani M., Katayama S. (1998). Dynamics of keyhole and molten pool in laser welding. J. Laser Appl..

